# First detection of bovine tuberculosis by Ziehl–Neelsen staining and polymerase chain reaction at dairy farms in the Lekok Sub-District, Pasuruan Regency, and Surabaya region, Indonesia

**DOI:** 10.14202/vetworld.2024.577-584

**Published:** 2024-03-08

**Authors:** Itfetania Aemilly Desire, Muhammad Luqman, Yulianna Puspitasari, Wiwiek Tyasningsih, Dhandy Koesoemo Wardhana, Dewa Ketut Meles, Yeni Dhamayanti, Dian Ayu Permatasari, Adiana Mutamsari Witaningrum, Agnes Dwi Sis Perwitasari, Hartanto Mulyo Raharjo, Siti Rani Ayuti, Shendy Canadya Kurniawan, Intan Noor Aina Kamaruzaman, Otto Sahat Martua Silaen

**Affiliations:** 1 Bachelor Program of Veterinary Medicine, Faculty of Veterinary Medicine, Universitas Airlangga, Surabaya, East Java, Indonesia; 2 Division of Veterinary Microbiology, Faculty of Veterinary Medicine, Universitas Airlangga, Surabaya, East Java, Indonesia; 3 Division of Veterinary Public Health, Faculty of Veterinary Medicine, Universitas Airlangga, Surabaya, East Java, Indonesia; 4 Division of Basic Veterinary Medicine, Faculty of Veterinary Medicine, Universitas Airlangga, Surabaya, East Java, Indonesia; 5 Division of Veterinary Anatomy, Faculty of Veterinary Medicine, Universitas Airlangga, Surabaya, East Java, Indonesia; 6 Department of Tuberculosis, Institute Tropical Disease, Universitas Airlangga, Surabaya, East Java, Indonesia; 7 Biochemistry Laboratory, Faculty of Veterinary Medicine, Universitas Syiah Kuala, Banda Aceh, Aceh, Indonesia; 8 Master Program of Animal Sciences, Department of Animal Sciences, Specialisation in Molecule, Cell and Organ Functioning, Wageningen University and Research, Wageningen, Netherlands; 9 Department of Veterinary Preclinical Sciences, Faculty of Veterinary Medicine, Universiti Malaysia Kelantan, Kelantan, Malaysia; 10 Doctoral Program of Biomedical Science, Faculty of Medicine, Universitas Indonesia, Jakarta, Indonesia

**Keywords:** bovine tuberculosis, cattle, polymerase chain reaction, public health, raw milk

## Abstract

**Background and Aim:**

Bovine tuberculosis (TB) is a zoonotic disease of great public health importance, particularly in Indonesia, where control measures are limited or are not implemented. This study aimed to detect the presence of *Mycobacterium* pathogens in milk samples from dairy cattle in Pasuruan regency and Surabaya City, East Java, using Ziehl–Neelsen acid-fast staining and polymerase chain reaction (PCR).

**Materials and Methods:**

Milk samples were aseptically collected from 50 cattle in the Lekok Subdistrict, Pasuruan Regency, and 44 from dairy farms in the Lakarsantri Subdistrict, Wonocolo Subdistrict, Mulyorejo Subdistrict, and Kenjeran Subdistrict, Surabaya, East Java. To detect Mycobacteria at the species level, each sample was assessed by Ziehl–Neelsen staining and PCR using the RD1 and RD4 genes.

**Results:**

The results of PCR assay from 50 samples in Lekok Subdistrict, Pasuruan Regency showed that 30 samples (60%) were positive for *Mycobacterium tuberculosis* and two samples (4%) were positive for *Mycobacterium bovis*, although Ziehl–Neelsen staining did not show the presence of *Mycobacterium* spp. In the Surabaya region, 31 samples (70.45%) were positive for *M. tuberculosis* and three samples (6.8%) were positive for *M. bovis*. Six samples (13.63%) from all PCR-positive samples could be detected microscopically with Ziehl–Neelsen.

**Conclusion:**

The presence of bovine TB in this study supports the importance of using a molecular tool alongside routine surveillance for a better understanding of the epidemiology of bovine TB in East Java.

## Introduction

Bovine tuberculosis (TB) is an infectious disease that has become widespread worldwide [1]. *Mycobacterium bovis* is a causative agent of bovine TB, which can infect farm animals, wild animals, and humans (zoonosis) [2]. *M. bovis* belongs to the *Mycobacterium tuberculosis* complex, which includes *M. tuberculosis, Mycobacterium africanum*, and *Mycobacterium microti* [3]. Detection of *Mycobacterium* infection in cattle samples is very important. Milk is a major source of protein and other nutrients that can be contaminated by pathogens and can transmit TB and other *Mycobacterium* infections from animals to humans [4]. Human and bovine varieties of TB are antigenically the same because cross-reactions can occur [1]. In the last two decades, *M. bovis* infection has infected humans in 0.5%–7.2% of all patients with a bacteriologically confirmed diagnosis of TB in developed countries, whereas in developing countries, *M. bovis* infection is still a major threat to public health [5]. The highest prevalence of TB in humans is found in the Asian region, where China, India, Bangladesh, Indonesia, and Pakistan together cause more than 50% of the global burden [6]. Two-thirds of the total global burden of TB is in eight countries: India (26%), Indonesia (8.5%), China (8.4%), Philippines (6.0%), Pakistan (5.7%), Nigeria (4.4%), Bangladesh (3.6%), and South Africa (3.6%) [7]. Although *M*. *bovis* infection, which causes TB in humans, is very low compared with *M*. *tuberculosis* infection, the very high risk among people who frequently interact with livestock must be considered [8]. Animals infected with *M. bovis* can potentially infect humans (zoonotic TB) [2]. *M. bovis* infection in animals and humans is a potential health risk [5].

Preventing zoonotic diseases by controlling and managing infections caused by *M. bovis* in cattle requires appropriate efforts [9]. Early detection of *M. bovis* infection in cattle is an initial step in prevention [10]. A tuberculin test on cattle can be performed as an initial step to detect TB [11]. Detection of TB lesions in slaughterhouses or farms should be followed by examination of the area of origin of the cattle to identify further cases [12]. The tuberculin test, which is useful for initial identification of TB in cattle, is only a measure of the presence of *M. tuberculosis* complex infection; therefore, it is necessary to perform a polymerase chain reaction (PCR) examination to determine whether it is *M. bovis* or another *Mycobacterium* spp. [11]. There is no information data or surveillance program on the spread of bovine TB in dairy cattle in East Java because the largest population of dairy cows is in East Java (approximately 296.3 thousand heads, or 50.68% of the total population of Indonesian dairy cows). Therefore, East Java may have the largest potential for TB cases in cattle [13]. East Java province has the second highest number of TB cases after West Java province [14]. The number of TB cases in East Java reached 41,404 cases, whereas those in West Java reached 62,563 cases [15]. Surabaya has the highest number of TB cases in East Java Province (3990 cases) [14]. Moreover, Pasuruan Regency is the largest contributor to the dairy cattle population in East Java Province and has the fifth largest number of TB cases in humans [16].

Zoonotic TB (*M. bovis*) transmission risk can occur in livestock, dairy cattle, and their products [17]. There is no definite information regarding the prevalence of zoonotic TB (*M. bovis* or *M. tuberculosis*) in cattle in Indonesia. The distinction between *M. bovis* and *M. tuberculosis* is still based on culture and biochemical methods. Although these methods are very difficult, time-consuming, and less accurate, PCR can be the best alternative method for accurately differentiating mycobacterial species [18].

This study aimed to detect bovine TB in cattle milk samples from Surabaya and Pasuruan Regency, East Java, using Ziehl–Neelsen acid-fast staining and PCR.

## Materials and Methods

### Ethical approval

All samples used in this study were obtained from routine milking activities performed by the farmer. All samples were in complete compliance with national regulations. Therefore, there was no need for ethical approval.

### Study period and location

The samples were collected from November 2022 to February 2023. Milk samples were collected from the Lekok sub-district in Pasuruan and four sub-districts in Surabaya (Lakarsantri, Wonocolo, Mulyorejo, and Kenjeran) of East Java, Indonesia.

### Sample collection

A total of 94 lactating cattle were used for milk sampling. Milk sampling was performed aseptically, and the samples were collected in a sterile storage bag. Thirty milliliters of milk sample was taken from each cattle. The samples were then centrifuged at 3000× *g* for 10 min. The pellets were used to prepare smears and extract DNA.

### Ziehl–Neelsen staining

Acid-fast bacilli (AFB) staining was performed using the Ziehl–Neelsen method, which provides high sensitivity and specificity and is a simple method of staining that was performed according to the manufacturer’s instructions using the Ziehl–Neelsen Acid-fast Bacillus Staining Kit (IndoReagen, Indonesia). The preparation was dried on a drying rack and then examined using a light microscope (Nikon, Japan) at 1000× magnification [19].

### Extraction of DNA from milk samples

DNA was extracted using a Wizard® Genomic DNA Purification Kit (Promega, USA) according to the manufacturer’s instructions. After all the extraction steps, the samples were incubated in a water bath at 65°C for 1 h for immediate use or incubated at 4°C for 24 h.

### Multiplex PCR for molecular detection

We used the procedure described by Sonekar *et al*. [20], with slight modifications, particularly in the initial denaturation time from 95°C for 7 min to 95°C for 2 min, and modifications in the annealing temperature from 59°C to 52°C for 1 min, as optimized in our study. The oligonucleotides used in this study were RD1 [21] and RD4 [22], with primary sequence RD1 (forward: CCC TTTCTCGTGTTTATACGTTTGA, reverse: GCCATATCGTCCGGAGCTT) and RD4 (forward: AATGGT TTGGTCATGACGCCTTC, reverse: CCCGTAGCG TTACTGAGAAATTGC). RD1 and RD4 were used to determine *Mycobacterium* spp., with reading of amplified PCR products at 110 bp and 176 bp positive for *M. bovis*, at 176 bp only positive for *M. bovis* BCG strain, and at 110 bp only positive for *M. tuberculosis* [20]. PCR reactions were performed in a total volume of 25 μL consisting of 12.5 μL GoTaq® Green Master Mix (Promega), 0.75 μL forward RD1, 0.75 μL reverse RD1, 0.5 μL forward RD4, 0.5 μL reverse RD4, 2 μL DNA template, and 8 μL nuclease-free water. A positive control was also included in the amplification analysis.

The PCR thermocycle for amplification was pre-denaturation at 95°C for 2 min, denaturation at 95°C for 1 min, annealing at 52°C for 1 min, extension at 72°C for 1 min, final extension at 72°C for 5 min, and cooling at 4°C for 5 min for 35 cycles. Amplification products (3 μL) were then analyzed by electrophoresis using a 100 bp DNA ladder (Promega) on 2% agarose gel (Invitrogen, USA) running at 90 V for 45 min. Subsequently, it was visualized by immersion in ethidium bromide fluorescence for 20 min, and the electrophoresis results were then viewed with an ultraviolet-transilluminator (ColeParmer, UK). The amplification product was declared positive for the RD1 gene if a single band is visible at 110 bp and the RD4 gene at 176 bp.

## Results

### Ziehl–Neelsen staining

**Figure-1:**
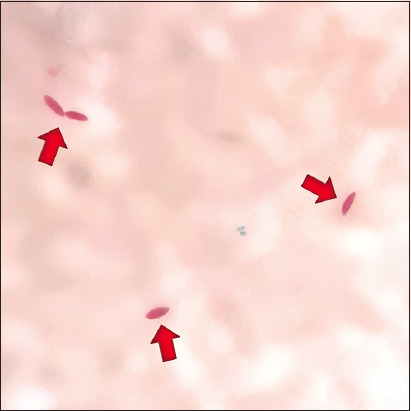
Microscopic examination of milk samples shows acid- fast bacilli of *Mycobacterium* spp. (red arrows) (Ziehl–Neelsen stain ×1000).

Six (13.63%) out of 44 dairy cows in Surabaya (Wonocolo sub-district and Kenjeran sub-district) were positive *for Mycobacterium* spp., whereas no *Mycobacterium* spp. was observed in any of the 50 samples from Lekok sub-district, Pasuruan (Table-1 and Figure-1).

**Table 1:** Data for Ziehl–Neelsen staining.

Location	Subdistrict	Number of samples	Number of positive Ziehl-Neelsen
Pasuruan	Lekok	50	0
Surabaya	Lakarsantri	7	1
	Wonocolo	22	1
	Mulyorejo	4	0
	Kenjeran	11	4
Total		94	6

### Detection using multiplex PCR

**Figure-2:**
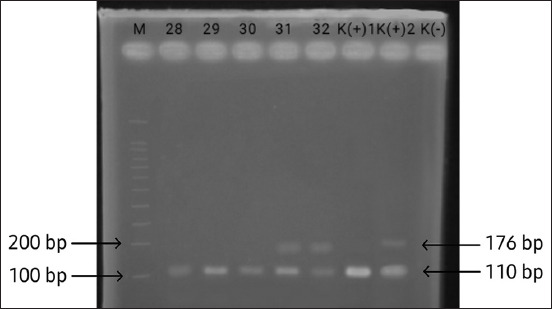
Electrophoresis of polymerase chain reaction products on a 2% agarose gel. M: Molecular size marker 100 bp; Lanes 28, 29, and 30 show a 110-bp fragment of the RD1 gene; Lanes 31, 32 show 110-bp and 176-bp fragments of the RD1 and RD4 genes; K (+)1 is a positive control for *Mycobacterium tuberculosis*; K(+)2 is a positive control for *Mycobacterium bovis*; K(–) is a negative control.

Of the 94 milk samples subjected to the PCR assay, 61 samples (64.89%) were positive for *M. tuberculosis* and 5 samples (5.31%) were positive for *M. bovis* (Table 2). DNA sample products were amplified at 110 bp (RD1) for M. *tuberculosis* and 110 bp + 176 bp (RD1 and RD4) for *M*. *bovis*. The results that appeared were considered positive for *M. tuberculosis* and showed one band above the 100 bp marker line, namely, 110 bp, whereas those positive for *M. bovis* showed two bands above the 100 bp marker line and below 200 bp, namely, 110 + 176 bp (Figure 2).

**Table 2:** PCR examination results.

Location	Subdistrict	Number of samples	PCR results	

			*M. tuberculosis*	*M. bovis*
Pasuruan	Lekok	50	30	2
Surabaya	Lakarsantri	7	5	0
	Wonocolo	22	16	1
	Mulyorejo	4	1	0
	Kenjeran	11	9	2
Total		94	61	5

PCR=Polymerase chain reaction,*** M. tuberculosis=Mycobacterium tuberculosis***, *M. bovis=Mycobacterium bovis*

## Discussion

*Mycobacterium* spp. cannot be classified as Gram-positive or Gram-negative because the cell wall of this bacterium does not have the characteristics of the outer membrane of Gram-negative bacteria [23]. This bacterium has a peptidoglycan–arabinogalactan–mycolic acid structure that acts as an external permeability barrier; therefore, when Gram-positive staining is performed, the color will appear very weak or not visible [24]. Using the Ziehl–Neelsen staining method, these bacteria appear red in color. The presence of *M*. *bovis* and *M. tuberculosis* with Ziehl–Neelsen staining is important to detect AFB [25]. One of the most commonly used microscopic methods in this study is AFB examination, which checks for *M. tuberculosis* or *M. bovis*.

The results of Ziehl–Neelsen staining and PCR were different. Fifty milk samples from the Lekok sub-district stained using the Ziehl–Neelsen method showed no presence of *Mycobacterium* spp. Three samples from Surabaya that showed positive results for *M. bovis* by PCR did not show results from Ziehl–Neelsen staining. Thirty-four milk samples from the Surabaya region were confirmed to be positive for *Mycobacterium* by PCR, and only six samples showed positive results by microscopic examination. This may be due to a small number of bacteria in the sample during the staining process. According to Holani *et al*. [26], many factors, including the prevalence of TB, the quality and number of specimens, coloring methods, and the ability of laboratory personnel to conduct the examination, can affect the sensitivity of staining with the Ziehl–Neelsen method.

AFB in samples containing at least 10,000 AFB/mL will be visible microscopically [27]. In Ziehl–Neelsen staining, to obtain clear color results, minimum sample handling must be performed for <2 h [28]. According to Bolaños *et al*. [4], AFB staining of milk has a low sensitivity; if the staining results are negative, it does not mean there is no infection in the sample. Sample handling and the number of bacterial specimens taken greatly determine the results in the Ziehl–Neelsen coloring method [29]. Another influencing factor in this method is the number of bacterial specimens taken, which makes it possible to take very few or even no bacteria, since the milk sample taken for the preparation is only one to two drops from the entire 10 mL sample.

In contrast to the PCR test, almost the entire 10 mL sample was processed to extract all the DNA, so the percentage of bacteria was quite large. Another study also explained that the examination of AFB in sputum using the Ziehl–Neelsen staining method will show a positive result if the sputum contains at least 10^5^ AFB/mL [26]. Microscopic examination results cannot distinguish *M. bovis* from other *Mycobacterium* spp. In the present study, PCR results were positive for *M. tuberculosis*. Ziehl–Neelsen staining can only show the presence of Mycobacterium but cannot determine its species [19]. *M. tuberculosis* and *M. bovis* infections are very difficult to distinguish based on clinical symptoms and pathological examination, as both show similar changes [30].

PCR is the most reliable technique for the rapid and specific detection of *Mycobacterium* spp. because it overcomes the lack of specificity of other traditional laboratory techniques, such as histopathology, and it allows the identification of *Mycobacterium* spp. from culture isolates or genomic DNA extracted from clinical specimens [31]. The PCR assay is the most convincing alternative approach for the rapid and specific diagnosis of TB [32]. It can detect the smallest trace of genetic material in a sample confirming exposure to the pathogen, does not require the isolation of the organism, and can detect DNA from both living and non-living organisms [20]. In the PCR test, even though the *M. tuberculosis* bacteria died, it was still detected because the DNA of the bacteria was detected [33].

PCR methods have been successfully used to diagnose bovine TB in various naturally infected clinical samples, such as tissue, blood, milk, and nasal exudates [34]. According to Cezar *et al*. [35], the PCR test detects TB with higher sensitivity than acid-resistant, fluorescent staining, and bacterial culture methods. This study identified *M. tuberculosis* and *M. bovis* using the primers RD1 and RD4. RD1 is one of the virulence factors containing the Early Secreted Antigenic Target 6 kDa gene, which is expressed by RD1 to be recognized by the immune response in the early stages of infection and is a potential immunostimulator [36]. RD4 is a flanking primer for specific deletions in the *M. bovis* genome and can be used to detect *M. bovis* AF2122/97, BCG Pasteur, and Veterinary Laboratory Agency panels of 10 different spoligotypes [37].

Samples were amplified at 110 and 176 bp. Bapat *et al*. [38] explained that using RD1 and RD4 primers amplified at 110 and 176 bp yielded specific results in detecting *M*. *tuberculosis* and *M*. *bovis* bacteria. This was confirmed by performing PCR tests on 85 goat blood samples suspected of TB infection and amplified at 110 and 176 bp. We found that 25 (33.33%) samples were positive for *M. bovis* and negative for *M. tuberculosis*. In total, 61 (64.89%) samples of *M. tuberculosis* and 5 (5.31%) samples of *M. bovis* from 94 samples collected from Surabaya City and Lekok sub-district, Pasuruan, were positive.

In this study, positive results for *M. tuberculosis* in 61 samples suggested zoonotic transmission of bacterial infection (human to animal). According to Chung *et al*. [39], age, socioeconomic conditions, environmental factors, community behavior factors, and a history of contact with patients with TB are factors associated with TB incidence. Factors that support the occurrence of infection in cattle include a very high frequency of contact with humans, a very close distance between cages and residential areas, and poor environmental conditions such as high humidity, poor ventilation, and poor feed conditions [40]. Transmission between cattle may also be influenced by the excretion frequency route of infection, infective dose, transmission period, and host susceptibility [41]. A study by Ramandinianto *et al*. [42] and Widodo *et al*. [43] reported that milk samples taken from individual dairy cows aseptically and placed into sterile plastic indicated microorganism contamination from human to cow milk.

The number of *M. tuberculosis* cases found in this study may be influenced by the frequency of contact with humans, the location of the farm in the middle of a residential area, the population density, and the high number of TB cases in this area. *M. tuberculosis* infection in farm animals (cattle) can potentially be transmitted back to humans [44]. *M. tuberculosis* does not have a native animal host or reservoir, and infected animals are likely to be accidental hosts. The main source of *M. tuberculosis* in animals, including cattle, is strongly believed to be humans with active TB [45]. According to Dwyer *et al*. [46], this can occur because *M. tuberculosis* and *M. bovis*, as the main hosts of specific pathogens, have the ability to infect a variety of hosts because they can cross species barriers, thereby spreading disease transmission between humans and animals.

Although the extent and risk of infection caused by *M. bovis* are unclear, *M*. *tuberculosis* can be isolated from tuberculous cattle, indicating a potential risk of transmission from cattle to humans [47]. *M. tuberculosis* infection occurs when a few airborne tubercle bacilli from a patient with active TB reach the host’s alveoli [48]. Because of such dispersal, the classical form of TB can develop in animals living in close contact with humans with active TB. Therefore, cross-transmission is a cause of the high positive yields of *M. tuberculosis* in the sample.

In this research, we delve into the intricacies of our findings regarding the detection methods employed for *Mycobacterium* spp., including the Ziehl–Neelsen staining method and PCR. We outline the differences observed between these two techniques and study the factors influencing the sensitivity of the Ziehl–Neelsen staining method. To our knowledge, which is supported by laboratory findings, revealed the PCR method has a greater sensitivity in detecting *Mycobacterium* spp. and is also able to differentiate between *M. tuberculosis* and *M. bovis* infections.

Understanding the potential zoonotic transmission of *M. tuberculosis* from cattle to humans is critical to establish ideal intervention/preventive strategies within the human and animal populations in Indonesia. Therefore, these findings serve as a future reference in elucidating the dynamics of tuberculosis transmission at the human-animal interface [49].

Moreover, through this study, we hope to expand its findings toward comprehensive analysis and its applications moving beyond mere summarization to offer insights that contribute to the advancement of knowledge in this field. In line with the emphasized zoonotic potential of *M. tuberculosis* and *M. bovis*, it is crucial to consider the human–animal interface and evaluate the characteristics of individuals involved in close contact with animals [17].

Understanding the demographics, occupational practices, and hygiene behaviors of animal husbandry workers can provide valuable insights into the potential pathways of zoonotic transmission [50]. Surveys or interviews with farm workers, veterinarians, and other relevant personnel could shed light on the risk factors associated with the transmission of TB between animals and humans [40]. Given the importance of molecular epidemiology in elucidating disease transmission dynamics, more in-depth molecular analysis of *Mycobacterium* isolates is required. Genomic sequencing or other molecular techniques can facilitate a comprehensive understanding of the genetic diversity, transmission patterns, and virulence factors of *Mycobacterium* strains that circulate within human and animal populations [51]. The molecular characterization of isolates can also help to distinguish between *Mycobacterium* spp. and identify potential reservoirs of infection [52]. Integrating these analyses into future research endeavors can enhance the ability to detect, monitor, and prevent zoonotic transmission of TB, ultimately contributing to more effective disease control strategies [53].

## Conclusion

There are cases of TB in cattle at dairy farms in Lekok Subdistrict, Pasuruan Regency, and Surabaya Regency that require further action; moreover, the study demands thorough testing of the flocks for continuous monitoring of TB to prevent its further spread. It stresses the need for further action and thorough testing of cattle herds in order to monitor and prevent the spread of TB. The findings of this study underscore the zoonotic potential of *M. tuberculosis* and *M. bovis*, highlighting the importance of evaluating the human–animal interface in the transmission of TB. To elucidate potential transmission pathways, further research efforts should focus on characterizing the demographics and behaviors of individuals in close contact with animals. In addition, a more extensive molecular analysis of *Mycobacterium* isolates is required to enhance our understanding of disease epidemiology and inform targeted control measures. If these knowledge gaps are addressed, the ability to mitigate the spread of TB between animals and humans can be improved, ultimately ensuring public health and animal welfare.

##  Authors’ Contributions

IAD and ML: Collected and assembled the data. SRA, SCK, and WT: Collected the samples, laboratory works, and drafted the manuscript. DKW and DKM: Analysis and interpretation of data. YD, OSMS, and DAP: Concept and design of the study. AMW, INAK, and ADSP: Investigation and data curation and reviewed the manuscript critically for important intellectual content. HMR and YP: Analysis and data curation and critically revised the manuscript. All authors have read, reviewed, and approved the final version of the manuscript.
